# Efficacy of anakinra in gouty arthritis: a retrospective study of 40 cases

**DOI:** 10.1186/ar4303

**Published:** 2013-09-17

**Authors:** Sébastien Ottaviani, Anna Moltó, Hang-Korng Ea, Séverine Neveu, Ghislaine Gill, Lauren Brunier, Elisabeth Palazzo, Olivier Meyer, Pascal Richette, Thomas Bardin, Yannick Allanore, Frédéric Lioté, Maxime Dougados, Philippe Dieudé

**Affiliations:** 1 Université Paris Diderot, Sorbonne Paris Cité, UFR de Médecine, F-75205 Paris, France; AP-HP, Service de Rhumatologie, Hôpital Bichat, 75018 Paris, France; 2 Université Paris Diderot, Sorbonne Paris Cité, UFR de Médecine, F-75205 Paris, France; AP-HP, Service de Rhumatologie, Pôle appareil Locomoteur, Hôpital Lariboisière, F-75475 Paris, France; 3 Université René Descartes, Service de Rhumatologie B, Hôpital Cochin, APHP, Paris, France; 4 Université René Descartes, Service de Rhumatologie A, Hôpital Cochin, APHP, Paris, France

**Keywords:** gout, IL-1, anakinra, arthritis

## Abstract

**Introduction:**

Gout is a common arthritis that occurs particularly in patients who frequently have associated comorbidities that limit the use of conventional therapies. The main mechanism of crystal-induced inflammation is interleukin-1 production by activation of the inflammasome. We aimed to evaluate the efficacy and tolerance of anakinra in gouty patients.

**Methods:**

We conducted a multicenter retrospective review of patients receiving anakinra for gouty arthritis. We reviewed the response to treatment, adverse events and relapses.

**Results:**

We examined data for 40 gouty patients (32 men; mean age 60.0 ± 13.9 years) receiving anakinra. Mean disease duration was 8.7 ± 8.7 years. All patients showed contraindications to and/or failure of at least two conventional therapies. Most (36; 90%) demonstrated good response to anakinra. Median pain on a 100-mm visual analog scale was rapidly decreased (73.5 (70.0 to 80.0) to 25.0 (20.0 to 32.5) mm, *P *<0.0001), as was median C-reactive protein (CRP) level (130.5 (55.8 to 238.8) to 16.0 (5.0 to 29.5) mg/l, *P *<0.0001). After a median follow-up of 7.0 (2.0 to 13.0) months, relapse occurred in 13 patients after a median delay of 15.0 (10.0 to 70.0) days. Seven infectious events, mainly with long-term use of anakinra, were noted.

**Conclusions:**

Anakinra may be efficient in gouty arthritis, is relatively well tolerated with short-term use, and could be a relevant option in managing gouty arthritis when conventional therapies are ineffective or contraindicated. Its long-term use could be limited by infectious complications.

## Introduction

Gout is a common arthritis caused by deposition of monosodium urate (MSU) crystals within and around joints secondary to chronic hyperuricemia. It affects 1% to 2% of adults in developed countries and may be increasing in prevalence [1]. Acute gouty arthritis may be associated with high inflammatory clinical and biological symptoms. Thus, one of the goals of management is rapid relief of inflammation [2,3].

Acute gouty attacks are usually treated with nonsteroidal anti-inflammatory drugs (NSAIDs), colchicine and corticosteroids [3]. Gouty patients often have concomitant renal, cardiovascular and gastrointestinal diseases as well as diabetes mellitus [4]. These comorbidities and associated treatments can lead to increased frequency of side effects or contraindications to conventional therapies for gouty arthritis [4]. We have abundant evidence of side effects from the use of colchicine (for example, for diarrhea) [5] and NSAIDs (for example, for gastrointestinal bleeding, cardiovascular events including myocardial infarction, renal impairment) [6,7], so care must be taken when prescribing such drugs. Thus, alternative therapies are needed for these 'difficult-to-treat' cases.

The main mechanism of crystal-induced inflammation is interleukin 1β (IL-1β) production by activation of the NLRP3 inflammasome [8], which strengthens the relevance of targeting IL-1β in patients with crystal-induced arthritis. Anti-IL-1 agents, such as anakinra, have been evaluated in gouty arthritis, for treating acute attacks or for preventing gouty attacks while initiating urate-lowering therapy [9-14]. To date, only two small open studies have evaluated the efficacy of anakinra in acute gouty arthritis [13,14] although anakinra has been labeled for rheumatoid arthritis treatment for more than 10 years. Other IL-1 inhibitors, canakinumab and rilonacept, appear to be effective in reducing pain and signs of inflammation in randomized controlled trials, which validate IL-1 as playing a pivotal role in gout inflammation [9,10,12,15].

Here, we aimed to evaluate the efficacy and safety of anakinra in patients with acute and chronic gouty arthritis but with contraindications to or failure of conventional therapies.

## Methods

### Patients

This was a multicenter retrospective review of charts for patients who received anakinra for gouty arthritis. Patients were identified by treating rheumatologists and by searching available electronic medical records with the keyword 'anakinra' or 'Kineret^®^'. Patients receiving anakinra who had concomitant connective tissue diseases were not included. Inclusion criteria were diagnosis of gouty arthritis defined as recommended by the identification of MSU crystals in synovial fluid [16] and at least one documented visit after the acute gouty arthritis requiring anakinra. The study was approved by the local institutional review board of Paris North Hospitals (No. 12-081) and all patients provided informed written consent to their physician to receive anakinra.

### Evaluation

We retrospectively assessed response to anakinra at baseline and at the first documented visit following the acute gouty arthritis according to the following items: swollen joint count (SJC) and tender joint count (TJC), patient evaluation of pain by a visual analog scale (VAS pain, 0 to 100 mm) and C-reactive protein (CRP) levels (mg/L). We collected data on demographics (age, gender), clinical variables (tophus, localization of arthritis, comorbid conditions, and disease and flare duration), radiologic features of gouty arthropathy and biological variables (serum uric acid levels (SUA), CRP and creatinine). The outcome of anakinra treatment was classified as good, partial, or no response. A good response was arbitrarily defined as an improvement >50% in VAS pain or CRP level and/or documentation in the chart of the word 'good' response after anakinra treatment. A partial response was defined as a report of improvement in joint symptoms but not a 'good' response (20% to 50% improvement). No response was defined as the absence of symptom relief (<20% improvement).

Adverse events were defined as diarrhea, myopathy and skin reactions with colchicine treatment; gastrointestinal bleeding, cardiovascular events, renal impairment and skin reactions with NSAIDs; hyperglycemia, hypertension and cardiovascular events with steroids; and local skin reaction, infection and neutropenia with anakinra.

Contraindications to conventional therapies and comorbidities were as described [4], except for osteoporosis and hyperlipidemia, which we did not consider a comorbidity limiting prescription of conventional therapies.

### Statistical analysis

Data are reported as mean ± SD or median (interquartile range (IQR)) or number (%). Non-parametric or Fisher's exact test was used to compare quantitative or categorical data, respectively. A two-tailed *P *<0.05 was considered statistically significant.

## Results

### Baseline characteristics

We investigated data for 40 patients (32 men) who received anakinra for gouty arthritis. Their baseline characteristics are shown in Table 1. In all, 79% and 92% of patients showed clinical tophi and gouty arthropathy, respectively.

**Table 1:** Baseline characteristics of patients receiving anakinra for gouty arthritis.

Baseline characteristics	Clinical characteristics of gouty patients
Number of patients	40
Number of men (%)	32 (80%)
Comorbid conditions (number; %)	HT (27; 68%), CKD 3-5 (22; 55%), CAD (17; 43%), AI (13; 33%), DM (9; 23%), GU (6; 15%), transplant (2 (kidney, heart); 5%), asthma (1; 3%),
Associated therapies (number; %)	LDA (8; 20%), oral anticoagulant (3; 8%),
Age, year, mean ± SD	60.0 ± 13.9
Disease duration, year, mean ± SD	8.7 ± 8.7
Flare duration, number patients (days, mean ± SD)	
Acute (<6 weeks)	34 (9.4 **± **8.1)
Subacute (6 to12 weeks)	2 (60.0 ± 1.4)
Chronic (>12 weeks)	4 (130 ± 45.8)
Localization of arthritis, (number; %)	Knees (30; 75%), wrists (22; 55%), ankles (24; 60%), MTP1s (20; 50%), MCPs (12; 30%), elbows (12; 30%), tarsae (6; 15%), shoulders (2; 5%)
Reason for anakinra use	
Non-response to conventional therapies, (number; %)	Colchicine (24; 60%), NSAIDs (11; 27.5%), steroids (7; 17.5%)
Adverse events or contraindication to conventional therapies, (number; %)	Colchicine (16; 40%), NSAIDs (29; 72.5%), steroids (9; 22.5%)

AI, alcohol intake; CAD, coronary heart disease; CKD, chronic kidney disease; DM, diabetes mellitus; GU, gastric ulcer; HT, hypertension, LDA, low-dose aspirin; MCPs, metacarpophalangeal joints; MTPs, metatarsophalangeal joints; NSAIDs, non-steroidal anti-inflammatory drugs.

At baseline, the median (IQR) pain level was 73.5 (70.0 to 80.0) mm. The median TJC and SJC was 5.0 (3.5 to 8.0) and 4.0 (3.0 to 5.5), respectively. The median CRP level was 130.5 (55.8 to 238.8) mg/L. The mean SUA was 534 ± 172 μM. In all, 17 (43%) patients had received urate-lowering therapy (allopurinol (*n *= 11), febuxostat (*n *= 6), benzbromarone (*n *= 1)). Diuretic drugs were prescribed for 14 patients (hydrochlorothiazides (*n *= 3), loop diuretics (*n *= 11)). All patients had a contraindication to or past history of adverse events with conventional treatments for acute gouty arthritis (Table 1).

The number of patients with gouty arthritis that was acute (<6 weeks), subacute (6 to 12 weeks) and chronic (>12 weeks) was 34, 2 and 4, respectively.

Among the 40 patients, 23 received anakinra following the protocol used by So *et al*.[14]: 100 mg daily for three days subcutaneously. Seven patients received anakinra for <15 days (100 mg/day: *n *= 6, 100 mg/2 days: *n *= 1). The 10 remaining patients received anakinra for the long term (>15 days), followed by a spacing of the dose regimen (median total duration: 5.0 (2.3 to 11.8) months).

### Anakinra response for gouty arthritis

#### Whole population

**Figure 1:**
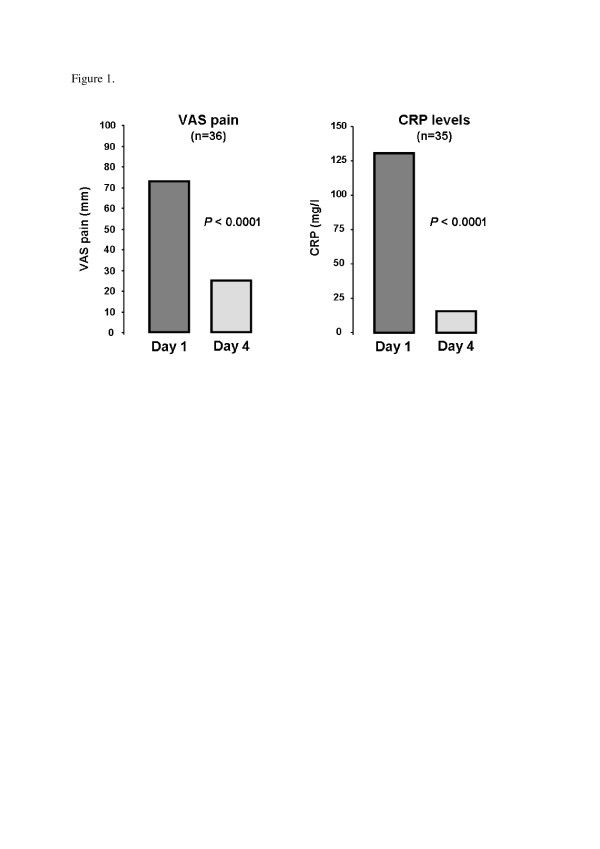
**Pain on a visual analog scale (VAS) and C-reactive protein level (CRP) on days 1 and 4 of anakinra treatment for gouty arthritis**. VAS, visual analog scale (mm); CRP, C-reactive protein (mg/l).

Of the 40 patients, good, partial and non-response to anakinra were noted in 36 (90%), 2 (5%) and 2 patients (5%), respectively. Pain score decreased from 73.5 (70.0 to 80.0) to 25.0 (20.0 to 32.5) mm, *P *<0.0001), as did CRP level (130.5 (55.8 to 238.8) to 16.0 (5.0 to 29.5) mg/L, *P *<0.0001) (Figure 1). In all, 30 patients received treatment to prevent relapse (Table 2). After a median follow-up of 7.0 (2.0 to 13.0) months, relapse occurred in 13 (32.5%) patients with a median delay of 15.0 (10.0 to 70.0) days. Relapse occurred particularly in patients not receiving therapy to prevent acute flare (7/10 versus 6/30, *P *= 0.006). No relapse occurred with long-term use of anakinra (>15 days).

**Table 2:** Response and follow-up of gouty arthritis patients receiving anakinra.

Follow-up characteristics	Anakinra for ≤3 days Number = 23	Anakinra for >3 days Number = 17
Response to anakinra, number (%)		
Good	20 (87%)	16 (94%)
Partial	2 (9%)	0 (0%)
No response	1 (4%)	1 (6%)
Follow-up duration, months, median (IQR)	6.0 (1.5 to 14.0)	8.0 (3.0 to 13.0)
Prevention of relapse, number (%)		
Total	17 (74%)^a^	13 (76%)
Low-dose colchicine	16 (89%)	7 (54%)
NSAIDs	3 (17%)	1 (8%)
Steroids	1 (6%)	2 (15%)
Anakinra	1 (6%)	9 (69%)
Relapse, number (%)	6 (26%)	7 (41%)
Delay to relapse, days, median (IQR)	15.0 (6.0 to 26.3)	60.0 (12.5 to 125.0)
Skin reaction	None	None
Infectious events under anakinra therapy (delay after starting anakinra)	H1N1 infection (1 day)	*Staphylococcus aureus *tophus (1 year) *S. aureus *tophus (4 years) *S. aureus *lung abcess (1 month) Erysipela of the leg (2 months) *Streptococcus B *urinary tract infection (1 month) *S. aureus *knee arthritis (1 year)

^a^Some patients also had different treatment. IQR, interquartile range; NSAIDs, non-steroidal anti-inflammatory drugs.

#### Anakinra response according to the dose regimen

A total of 23 patients received anakinra, 100 mg/day, for up to three days, with good response in 20 (87%); 17 (74%) showed relapse prevention after resolution of the flare. After a median follow-up of 6.0 (1.5 to 14.0) months, relapse occurred in six (26%) patients at a median delay of 15.0 (6.0 to 26.3) days.

In all, 17 patients received anakinra for more than three days, with good response in 16 (94%); 13 (76%) showed relapse prevention after flare resolution. After a median follow-up of 8.0 (3.0 to 13.0) months, relapse occurred in seven (41%) patients at a median delay of 60.0 (12.5 to 125.0) days.

### Tolerance

No patient reported anakinra-related skin hypersensitivity. A total of seven infectious complications, mainly staphylococcal infections, were reported in six patients (Table 2). One H1N1 viral infection occurred one day after anakinra was started (previously reported in [17]). All other infectious complications occurred in patients with long-term use of anakinra and were successfully treated with antibiotics. Of the six patients, five restarted anakinra after the resolution of infection. No patient has shown tuberculosis or pneumococcal infection.

## Discussion

Recently, the emerging role of IL-1β in the pathogenesis of inflammation in crystal-induced arthritis [8,18,19] led to considering anti-IL-1 therapies as a relevant alternative to conventional therapies for gouty arthritis. Here, we report on our experience with anakinra therapy, a recombinant receptor antagonist against IL-R blocking both IL-1β and β, for gouty arthritis in a large series of patients. Our results are in good agreement with those of the first open-label trial of anakinra showing improved patient-reported symptoms by at least 50% of all 10 patients enrolled [14]. Chen *et al*. suggested an efficiency in 6 of 10 patients with good response [13]. Similar to So *et al*., we observed a rapid decrease of both VAS pain and CRP levels. Recently, randomized controlled trials found that two anti-IL-1β biologic agents (rilonacept and canakinumab) prevented gouty arthritis during the initiation of urate-lowering therapy with allopurinol [10,11], but only canakinumab demonstrated efficacy for acute gouty arthritis [9,12]. In these cases, rilonacept failed to induce a rapid relief of symptoms [20] but could decrease pain in chronic gouty arthritis [15]. These data strengthen the argument to target IL-1 blockade for acute gouty arthritis. Of interest, canakinumab recently obtained European authorization [21].

In our study, relapse occurred frequently among patients not receiving therapy to prevent acute flare, and conversely, no patient with long-term use of anakinra experienced relapse. The daily subcutaneous injection could limit the use of the drug in preventing flare, although skin tolerance was excellent in our study. The short-term regimen was well tolerated: only one viral infection was observed. This short-term good tolerance agrees with previous studies of anakinra for gouty arthritis [13,14] and pseudogout [22,23]. However, long-term use was poorly tolerated, with six infectious events, notably one septic arthritis and one patient with pulmonary abscesses. These data suggest a risk of infection with prolonged administration. To date, the role IL-1 antagonists could play in clinical practice is unclear. Nonetheless, their cost (38€ per injection in France, 2013) may not be excessive in managing acute attacks, particularly in patients with contraindications to, or who cannot tolerate, conventional therapies. Recently, American College of Rheumatology recommendations allow for use of anti-IL1 agents when conventional therapies have failed or are contraindicated [2]. Of note, anakinra, with the shortest half-life of the IL-1 blockers, could be a relevant option to manage acute gouty arthritis.

Our study had some limitations. First, data were retrospectively collected, with varied use of anakinra, and we had no control group. However, this real-life observational study is the largest reported series for this treatment. Randomized controlled studies are necessary to clarify the place of anakinra in the management of gouty arthritis flare.

## Conclusions

Our results provide evidence that anakinra is effective, relatively well tolerated with short-term use, and could be a good alternative for treating gouty arthritis in patients for whom conventional therapies are ineffective or contraindicated. Although these findings are promising, this was a retrospective study, and future randomized controlled trials are required definitely to determine the place of anakinra in managing gouty arthritis.

## Abbreviations

CRP: C-reactive protein; IL-1: interleukin-1; IQR: interquartile range; MSU: monosodium urate; NSAIDs: nonsteroidal anti-inflammatory drugs; SD: standard deviation; SJC: swollen joint count; SUA: serum uric acid; TJC: tender joint count; VAS: visual analog scale.

## Competing interests

Frédéric Lioté received unrestricted grants from SOBI France and Novartis since 2010 for helping set up an annual European Workshop on crystal-induced inflammation and human diseases. The other authors declare they have no competing interests.

## Authors' contribution

SO and PD conceived of the study, made substantial contributions to the acquisition of data, participated in its design, and wrote the manuscript. AM, HKE, SN, GG, LB, EP, PR, TB, YA, FL, MD and OM made substantial contributions to the acquisition of data and helped to draft the manuscript. All authors read and approved the final manuscript.

## References

[B1] Richette P Bardin T Gout Lancet 2009 15 318 328 19692116 10.1016/S0140-6736(09)60883-7

[B2] Khanna D Khanna PP Fitzgerald JD Singh MK Bae S Neogi T Pillinger MH Merill J Lee S Prakash S Kaldas M Gogia M Perez-Ruiz F Taylor W Liote F Choi H Singh JA Dalbeth N Kaplan S Niyyar V Jones D Yarows SA Roessler B Kerr G King C Levy G Furst DE Edwards NL Mandell B Schumacher HR 2012 American College of Rheumatology guidelines for management of gout. Part 2: therapy and anti-inflammatory prophylaxis of acute gouty arthritis Arthritis Care Res (Hoboken) 2012 15 1447 1461 23024029 10.1002/acr.21773 PMC3662546

[B3] Zhang W Doherty M Bardin T Pascual E Barskova V Conaghan P Gerster J Jacobs J Leeb B Liote F McCarthy G Netter P Nuki G Perez-Ruiz F Pignone A Pimentao J Punzi L Roddy E Uhlig T Zimmermann-Gorska I EULAR evidence based recommendations for gout. Part II: management. Report of a task force of the EULAR Standing Committee for International Clinical Studies Including Therapeutics (ESCISIT) Ann Rheum Dis 2006 15 1312 1324 10.1136/ard.2006.055269 16707532 PMC1798308

[B4] Keenan RT O'Brien WR Lee KH Crittenden DB Fisher MC Goldfarb DS Krasnokutsky S Oh C Pillinger MH Prevalence of contraindications and prescription of pharmacologic therapies for gout Am J Med 2011 15 155 163 10.1016/j.amjmed.2010.09.012 21295195

[B5] Terkeltaub RA Colchicine update: 2008 Semin Arthritis Rheum 2009 15 411 419 10.1016/j.semarthrit.2008.08.006 18973929

[B6] Park SC Chun HJ Kang CD Sul D Prevention and management of non-steroidal anti-inflammatory drugs-induced small intestinal injury World J Gastroenterol 2011 15 4647 4653 10.3748/wjg.v17.i42.4647 22180706 PMC3237301

[B7] Brater DC Anti-inflammatory agents and renal function Semin Arthritis Rheum 2002 15 33 42 10.1053/sarh.2002.37216 12528072

[B8] Martinon F Petrilli V Mayor A Tardivel A Tschopp J Gout-associated uric acid crystals activate the NALP3 inflammasome Nature 2006 15 237 241 10.1038/nature04516 16407889

[B9] So A De Meulemeester M Pikhlak A Yucel AE Richard D Murphy V Arulmani U Sallstig P Schlesinger N Canakinumab for the treatment of acute flares in difficult-to-treat gouty arthritis: results of a multicenter, phase II, dose-ranging study Arthritis Rheum 2010 15 3064 3076 10.1002/art.27600 20533546

[B10] Schumacher HR Jr Evans RR Saag KG Clower J Jennings W Weinstein SP Yancopoulos GD Wang J Terkeltaub R Rilonacept (interleukin-1 trap) for prevention of gout flares during initiation of uric acid-lowering therapy: results from a phase III randomized, double-blind, placebo-controlled, confirmatory efficacy study Arthritis Care Res (Hoboken) 2012 15 1462 1470 10.1002/acr.21690 22549879

[B11] Schlesinger N Mysler E Lin HY De Meulemeester M Rovensky J Arulmani U Balfour A Krammer G Sallstig P So A Canakinumab reduces the risk of acute gouty arthritis flares during initiation of allopurinol treatment: results of a double-blind, randomised study Ann Rheum Dis 2011 15 1264 1271 10.1136/ard.2010.144063 21540198 PMC3103669

[B12] Schlesinger N Alten RE Bardin T Schumacher HR Bloch M Gimona A Krammer G Murphy V Richard D So AK Canakinumab for acute gouty arthritis in patients with limited treatment options: results from two randomised, multicentre, active-controlled, double-blind trials and their initial extensions Ann Rheum Dis 2012 15 1839 1848 10.1136/annrheumdis-2011-200908 22586173

[B13] Chen K Fields T Mancuso CA Bass AR Vasanth L Anakinra's efficacy is variable in refractory gout: report of ten cases Semin Arthritis Rheum 2010 15 210 214 10.1016/j.semarthrit.2010.03.001 20494407

[B14] So A De Smedt T Revaz S Tschopp J A pilot study of IL-1 inhibition by anakinra in acute gout Arthritis Res Ther 2007 15 R28 10.1186/ar2143 17352828 PMC1906806

[B15] Terkeltaub R Sundy JS Schumacher HR Murphy F Bookbinder S Biedermann S Wu R Mellis S Radin A The interleukin 1 inhibitor rilonacept in treatment of chronic gouty arthritis: results of a placebo-controlled, monosequence crossover, non-randomised, single-blind pilot study Ann Rheum Dis 2009 15 1613 1617 10.1136/ard.2009.108936 19635719 PMC2732898

[B16] Zhang W Doherty M Pascual E Bardin T Barskova V Conaghan P Gerster J Jacobs J Leeb B Liote F McCarthy G Netter P Nuki G Perez-Ruiz F Pignone A Pimentao J Punzi L Roddy E Uhlig T Zimmermann-Gorska I EULAR evidence based recommendations for gout. Part I: diagnosis. Report of a task force of the Standing Committee for International Clinical Studies Including Therapeutics (ESCISIT) Ann Rheum Dis 2006 15 1301 1311 10.1136/ard.2006.055251 16707533 PMC1798330

[B17] Nocturne G Ora J Ea HK Liote F Influenza A H1N1 and anakinra exposure in a patient with gout Joint Bone Spine 2010 15 369 370 10.1016/j.jbspin.2010.04.005 20554240

[B18] Gross O Yazdi AS Thomas CJ Masin M Heinz LX Guarda G Quadroni M Drexler SK Tschopp J Inflammasome activators induce interleukin-1alpha secretion via distinct pathways with differential requirement for the protease function of caspase-1 Immunity 2012 15 388 400 10.1016/j.immuni.2012.01.018 22444631

[B19] Church LD Cook GP McDermott MF Primer: inflammasomes and interleukin 1beta in inflammatory disorders Nat Clin Pract Rheumatol 2008 15 34 42 18172447 10.1038/ncprheum0681

[B20] Terkeltaub RA Schumacher HR Carter JD Baraf HS Evans RR Wang J King-Davis S Weinstein SP Rilonacept in the treatment of acute gouty arthritis: a randomized, controlled clinical trial using indomethacin as the active comparator Arthritis Res Ther 2013 15 R25 10.1186/ar4159 23375025 PMC3672764

[B21] Euopean Medicines Agency Ilaris 2013 http://www.ema.europa.eu/docs/fr_FR/document_library/EPAR_-_Summary_for_the_public/human/001109/WC500031677.pdf

[B22] Molto A Ea HK Richette P Bardin T Liote F Efficacy of anakinra for refractory acute calcium pyrophosphate crystal arthritis Joint Bone Spine 2012 15 621 623 10.1016/j.jbspin.2012.01.010 22658375

[B23] Ottaviani S Brunier L Sibilia J Maurier F Ardizzone M Wendling D Gill G Palazzo E Meyer O Dieude P Efficacy of anakinra in calcium pyrophosphate crystal-induced arthritis: a report of 16 cases and review of the literature Joint Bone Spine 2013 15 178 182 10.1016/j.jbspin.2012.07.018 23022422

